# Industrial Application of Nanocelluloses in Papermaking: A Review of Challenges, Technical Solutions, and Market Perspectives

**DOI:** 10.3390/molecules25030526

**Published:** 2020-01-25

**Authors:** Ana Balea, Elena Fuente, M. Concepcion Monte, Noemi Merayo, Cristina Campano, Carlos Negro, Angeles Blanco

**Affiliations:** 1 Department of Chemical Engineering and Materials, Universidad Complutense de Madrid (UCM), Av. Complutense s/n, 28040 Madrid, Spain; anabalea@ucm.es (A.B.); helenafg@ucm.es (E.F.); cmonte@ucm.es (M.C.M.); nmerayoc@ucm.es (N.M.); ccampano@ucm.es (C.C.); cnegro@ucm.es (C.N.); 2 Department of Mechanical, Chemical and Industrial Design Engineering, ETSIDI, Universidad Politécnica de Madrid (UPM), Ronda de Valencia 3, 28012 Madrid, Spain

**Keywords:** industrial nanocellulose use, cellulose nanofibers, cellulose microfibers, cellulose nanocrystals, paper quality

## Abstract

Nanocelluloses (NC) increase mechanical and barrier paper properties allowing the use of paper in applications actually covered by other materials. Despite the exponential increase of information, NC have not been fully implemented in papermaking yet, due to the challenges of using NC. This paper provides a review of the main new findings and emerging possibilities in this field by focusing mainly on: (i) Decoupling the effects of NC on wet-end and paper properties by using synergies with retention aids, chemical modification, or filler preflocculation; (ii) challenges and solutions related to the incorporation of NC in the pulp suspension and its effects on barrier properties; and (iii) characterization needs of NC at an industrial scale. The paper also includes the market perspectives. It is concluded that to solve these challenges specific solutions are required for each paper product and process, being the wet-end optimization the key to decouple NC effects on drainage and paper properties. Furthermore, the effect of NC on recyclability must also be taken into account to reach a compromise solution. This review helps readers find upscale options for using NC in papermaking and identify further research needs within this field.

## 1. Introduction

Paper is a biodegradable material with a high potential to replace plastic in the production of packages and bags. However, the papermaking industry is continuously challenged by different aspects: (i) The increasing requirements of mechanical, physical, and printing properties to accomplish the high quality demand of paper products, (ii) the deterioration of the recycled fibers as a consequence of the increasing recycling rate, and (iii) the restrictions in the production costs [1,2].

The use of nanocelluloses (NC) in papermaking can contribute significantly to improve paper quality [3]. NC present several advantages which includes a high surface area, unique optical properties, lightweight, stiffness, high strength, etc. In addition, their inherent properties related to cellulose, such as biodegradability, renewability and sustainability, have attracted a high interest for both researchers and industries. All these aspects and the NC compatibility with the pulp, make NC a smart product and a potential solution for many of the challenges of the papermaking industry. Nowadays, many successful results have been published about different applications of NC in paper, including several review articles and book chapters that analyze the potential uses of NC in this field [4,5,6,7,8,9,10,11,12,13]. Among the different uses of NC in papermaking, their application as dry strength additive [13] and coating agent [6] are predominant. Nevertheless, other approaches have been considered, such as their use as wet strength aid [14,15], as a retention additive [16,17], for flexographic inks removal [18,19], for book restauration [20], as linting control agent [21,22] or as a vehicle to confer special paper properties, such as antimicrobial [23], electric behavior [24] and fireproof [25]. However, due to the high production costs of NC, they have not been fully commercialized at industrial scale.

Many terms have been used in the past to distinguish the different types of NC (e.g., microfibrillated cellulose, nanofibrillated cellulose, nanofibers, cellulose nanowhiskers, nanorods, microbial cellulose, etc.) [26,27]; but the ISO/TS 20477:2017 standard has unified the terminology. Cellulose nanofibers (CNF), cellulose nanocrystals (CNC), and bacterial cellulose (BC) are the three main types of NC that differ in their dimensions, properties, functions, and preparation methods. The production of CNF requires breaking the hydrogen bonds to separate the fibers into nanofibrils. CNF are obtained mainly by mechanical treatments of the pulp, including homogenization at high pressure, microfluidization, grinding, electrospinning, steam explosion, among others [28,29]. These processes are frequently highly expensive, so typically chemical or enzymatic pretreatments are carried out to facilitate the separation of fibers, increasing the CNF yield and reducing the costs [30]. On the other hand, CNC have been traditionally produced by acid hydrolysis [31]. High hydrolysis yield in CNC production requires pure cellulose or, at least, a raw material rich on cellulose. Some cellulose sources, such as cotton, BC, and microcrystalline cellulose (MCC), are composed essentially by cellulose, so direct acid hydrolysis can be conducted. However, when wood or other biomass sources are used, components such as extractives, hemicelluloses, lignin, and inorganic particles, need to be removed by different pretreatments. These pretreatments usually include a first extraction with dichloromethane, acetone, or ethanol/benzene [32], an alkali treatment [33], a kraft cooking process [34] and finally, several bleaching cycles [33]. All these treatments not only decrease considerably the process yield, but also increase the production costs and the environmental impact. However, high purity does not always mean a high NC performance [35]. In the case of BC, the high amount of nutrients needed to culture the acetobacteria, together with the slow rate of BC production, triggers a highly expensive process. When bacteria are cultured in static mode, the BC is produced in the form of highly and strongly entangled nanofibrils, mainly due to the natural movement of bacteria towards the surface in search of oxygen [36]. These BC pellicles present a high difficulty in dispersing, [37,38], but they are useful for giving special properties to specific paper products, for example, fire resistant paper, electronic and magnetic paper [8,25,39].

Industrial scale application of NC in papermaking is just starting and it is limited by the risks and costs. Therefore, further research and development is needed to address various cost related issues associated with its production, its characterization, and the variables that influence their application at industrial scale. This review aims to compile and discuss the technical challenges presented when NC is used in papermaking. Moreover, different emerging possibilities have been reviewed in the literature to facilitate their industrial implementation, such as the reduction of water permeability to improve the barrier properties of NC, the enhancement of the dispersion of NC in the pulp or over the wet web to improve the paper properties, the decoupling of retention and drainage effects, and the reduction of the NC costs, among others. Furthermore, the main challenges in NC characterization and a market perspective of NC is also presented.

## 2. Challenges and Solutions of Industrial Application of Nanocelluloses in Papermaking

In papermaking, NC could be mixed with the pulp before paper formation or used as coating in the wet-sheet before drying. The improvements in tensile strength, burst index, and internal cohesion of paper with NC are due to the high number of hydroxyl groups present on the active surface of the NC, which increases the hydrogen bonding with the fibers. Thus, when the aim of using NC is to increase the mechanical properties of paper products, they are usually mixed with the pulp. However, this high hydrogen bonding ability could increase the energy consumption for an efficient NC dispersion, decrease brightness, and increase the drainage time of the pulp [40,41]. Moreover, the efficient retention of NC within the fiber network, their interaction with the retention system, and the increase in energy consumption during drying must be optimized to scale up NC at industrial level.

The addition of NC by coating the web-paper eliminates some of the problems described above but other challenges appear [42], for example, its homogeneous distribution [43,44] and its attachment to the paper [45].

In addition, NC have other drawbacks related to their inherent properties, mainly their nanometric size and their high swelling ability which have an impact on porosity and barrier properties, respectively. The presence of NC on paper decreases porosity which increases drainage time decreasing the productivity of the paper machine. The presence of CNF can increase Schopper–Riegler up to 200% because of the lower porosity of the wet web [46]. Low porosity improves barrier properties, which is interesting in packaging applications, but the high CNF swelling ability increases water vapor permeability of papers affecting barrier properties [47]. 

### 2.1. Bulk Addition of Nanocellulose

The optimization of the wet-end by the selection of the best retention additives for each NC, as well as the correct dose and addition points, can decouple retention and drainage effects [48]. In addition, when mineral fillers are preflocculated by NC, the fillers and NC retention, as well as the paper mechanical properties can be improved. The modification of NC could be an opportunity to further increase their bonding capacity, control the interactions with fibers and fillers, and reduce the consumption of retention agents. Finally, the adequate NC dispersion and mixing within the furnish are also key points to enhance paper mechanical properties when bulk addition of NC is carried out.

#### 2.1.1. Wet-End Optimization

The nanometric size of NC and their anionic charge make them difficult to be retained within the paper matrix [49,50]. On the other hand, NC retention into the fiber network usually causes a detrimental effect on the drainage rate, since the wet-web porosity is strongly reduced and the water binding is much greater [49,51,52]. Taipale et al. [51] and Hii et al. [53] observed that the drainage time increased proportionally to the CNF dosage because the CNF retention was favored, thus blocking the pores of the sheets. Espinosa et al. [54] explained the effect on drainage in terms of increased water retention ability of mechanically microfibrillated cellulose, which increased viscosity of the suspension. They compared the effects on drainage caused by the addition of different types and doses of NC with those due to the pulp refining [54]. They observed that getting the target breaking length for recycled cardboard (3443 m) required the use of 1000 rev of mechanical refining or the addition of 1.5% CNF. If mechanically ultrafine grinder CNF from wheat straw were used, the drainage time did not increase.

Although initial studies report the negative effect of NC on drainage, it can be kept or even improved when the adequate combination of retention system (RS) and NC are used, as shown in Table 1.

Over the past thirty years, dual retention systems based on the combination of polymers and microparticles have been developed to control chemical flocculation and optimize retention, drainage, and formation [55,56]. The microparticle is usually anionic bentonite or microsilica. Recently, several authors have observed that the use of CNF as a microparticle, in combination with a polyelectrolyte, such as polyacrylamide (PAM), improves the retention of fillers and the mechanical properties without a detrimental effect on drainage (Table 1) [35,41,57]. Petroudy et al. [58] showed that it is possible to achieve a high tensile index (TI) increment by combining CNF and cationic PAM (CPAM) without increasing the drainage time. Merayo et al. [41] also proved that both TI and drainage rate can be enhanced by using 1.5 wt% CNF with a dual retention system composed of CPAM and bentonite. Moreover, with the use of chitosan and 1 wt% CNF, the drainage rate was increased by 50% and the paper sheets achieved a TI improvement of 16% and 14% when CNF are from bleached *Eucalyptus* Kraft pulp and corn stalk organosolv pulp, respectively. Lourenço et al. [57] found that the oxidation degree of CNF was closed related to their effect on filler retention. The excess of oxidation led to a CNF with too high carboxylic content and too low polymerization degree with low filler flocculation ability and with high affinity to CPAM, forming CPAM-CNF bundles that were not able to attach the filler to the fibers. Those CNF were obtained by means of oxidation catalyzed by 2,2,6,6-Tetramethylpiperidin-1-yl)oxyl (TEMPO). TEMPO-mediated oxidation increases the anionic charge of the cellulose surface and the electrostatic repulsion among the fibrils. This contributes to fibrillation decreasing the diameter and increasing available surface for interactions. In this work by Lourenço et al. [57], the CNF diameter for two different intensities of TEMPO-mediated oxidation was estimated from their specific surface area and assessed by AFM. However, due to the broad diameter distribution, differences in diameter were not considered significant enough to explain differences in retention efficiency and discussion was based in length differences, which are clearly higher. Several authors [59,60] observed that the CNF diameter decreased notably by increasing TEMPO-mediated oxidation, but the effect is asymptotic and very low further decreases were observed when the amount of NaClO used was over 10 mmol/g. The specific surface area and swelling ability increased in the same way than the diameter decreased. This affects the drainage since interaction of CNF with water increases.

Xu et al. [65] studied the performance of CNC in combination with CPAM and cationic starch (CS). In both cases, when the addition of CNC was higher than 0.6 wt%, the first pass retention reached its highest level (90.5% for CPAM/CNC and 88.5% for CS/CNC) while in absence of CNC it was around 87%. Moreover, when the CNC was used together with CPAM and CS, the pulp drainage was not deteriorated and the strength of the properties were further improved.

Lenze et al. [66] proved that the efficiency on retention and drainage of dual retention systems comprising CNC as anionic nanoparticle depended on the CNC length. They prepared three different CNC by means of cryo-crushing either dry (with a length of 63 nm) or wet CNC (lengths of 80 and 103 nm). Handsheets were prepared from 100% recycled copy paper in dilute Na_2_SO_4_, with poly-DADMAC as coagulant, and a very-high-mass cationic acrylamide copolymer combined with the CNC as retention system. The dual retention system with the uncrushed CNC was the most effective in improving fine-particle retention and promoting dewatering. This is due to formation of longer bridges between solid surfaces in the suspension by means of the uncrushed CNC.

On the other hand, it is important to notice that drainage time is strongly influenced by the prevailing conditions, such as pH, salt concentration, type of cationic polyelectrolyte, and beating level of the pulp [51]. At low pH, the carboxylic groups of TEMPO-oxidized CNF are protonated, which decreases the repulsive forces among them and reduces the water retention capacity of the network, thus enhancing drainage. The contribution of salt concentration is more complex: The minimum drainage rate was found at a salt concentration of 0.01 M. At a lower salt concentration, the swelling of NC and fibers is increased due to the osmotic pressure change. Moreover, the electrostatic forces decrease with higher salt concentration affecting flocculation. The double electrostatic layer thickness decreases with increasing ionic strength. This can improve flocculation, but it affects sheet formation [51].

Amorphous regions of CNF hold a higher water amount than crystalline domains, which restricts even more the drainage rate. Thus, with the removal of amorphous cellulose in CNC, the negative effect on the drainage rate may be reduced. As observed by Verma et al. [67], the enzymatic hydrolysis of cellulose with endoglucanase improves the pulp drainability by 11%–25%, as well as paper strength and smoothness. Lenze et al. [66] also observed that the effect of CNC on drainage depends on their length. They concluded that intact CNC (103 > 63 nm), were more effective to fine-particle retention, promoting at the same time the dewatering. In addition, the charge of the CNC was also demonstrated to influence on the release of water from cellulosic paper [68].

#### 2.1.2. Preflocculation of Fillers Induced by Nanocelluloses

Linear polymers can change significantly their conformation with time, shearing and concentration, while the CNF and derivatives, although being flexible, have a more stable conformation than that of polymers and they are expected to behave in a similar way to highly cross-linked polyelectrolytes, forming reversible flocs. However, the interaction of CNF with particles is favored by their higher size compared to polyelectrolytes [48]. This explains the results obtained by He and Hwang [69] and by Lourenço et al. [57], who showed the efficient use of CNF as a flocculant for cationic fillers, such as precipitated calcium carbonate (PCC). In addition, the system had a high reflocculation capability, also studied by Korhonen and Laine [63].

Some authors have studied the feasibility of adding CNF or CNC to the filler suspension before mixing with the pulp. The NC interacts with the fillers producing a filler-NC complex, usually by means of adsorbing NC on the mineral particles. He et al. [70] formed a complex PCC-CS-CNF and reached improvements in both TI (from 26 to 42 Nm/g) and PCC retention (from 85% to 92%) compared to those obtained by the addition of each component to the pulp separately. Moreover, although large flocs were observed in the complex suspension, the formation and the opacity of the sheet were also improved. They observed that the CNF glued PCC particles, improving the interaction of PCC with the fiber network and increasing the number of small sized optically active pores. This mechanism was deeply studied by Lourenço et al. [57]. They proposed that the CNF flocculated the PCC particles by patching, taking into account the low CNF polymerization degree. The flocs were attached to the fiber surface by bridging driven by CPAM. They considered the possibility that some CNF formed bridges between fibers and PCC-CNF flocs too.

Ottesen et al. [71] tried to mix the CNF, prepared by grinding softwood pulp, with ground calcium carbonate (GCC) previous to their addition to the furnish. They proved that pre-flocculating the fillers with both CNF and CPAM/bentonite dual retention system before adding to the pulp improved paper mechanical properties and reduced the negative effect of CNF on dewatering. The adsorption of CNF on fibers increased their specific surface area, but the adsorption of CNF on GCC also modified their surface chemistry. Therefore, the interaction of GCC with the fibers, fines, and retention system was improved, enhancing the mechanical properties of the sheet.

Lourenço et al. [61] proposed the use of enzymatic cellulose microfibers instead of TEMPO-CNF for PCC preflocculation. They proved that PCC floc size increased with polymerization degree of the enzymatic cellulose microfibers. They demonstrated that the use of these microfibers can improve the PCC retention and both the paper dry and wet tensile strengths, even without any other retention agents, providing the right enzyme cocktail was selected. This allows saving flocculant costs.

Tarres et al. [59] compared the properties of enzymatic CNF and TEMPO mediated oxidation. The CNF obtained by enzymatic pretreatment had a lower fibrillation and surface, and a higher diameter than that obtained by TEMPO mediated oxidation. However, the polymerization degree and length were larger, which contribute to explain the improvement in filler retention.

Rantanen et al. [72] went a step over and attached PCC on the surface of CNF and microfibrillated cellulose. This strategy improved significantly the drainage step and the paper opacity, young modulus, and TI, which contained up to 70% of fillers.

#### 2.1.3. Modification of Nanocellulose

In order to improve the retention of either CNF or CNC, the modification of the surface charge density has been considered by different authors. Generally, a chemical pretreatment of NC during production or by means of NC reaction with a convenient reagent are proposed (Table 2).

Since retention systems are always cationic, to allow them to attach the fillers with the fibers and fines, the approach of NC cationizing could make them feasible to be used as a retention agent. It can avoid or minimize the requirements of other synthetic retention systems and flocculants. This would make the process more cost-efficient by improving both the retention of NC and mineral fillers and the mechanical properties [81]. There are numerous methods to cationize NC, as shown in Table 2.

Xiang et al. [78] cationized BC fibers by reaction with CHPTAC with a low degree of substitution (0.005), reaching an extra 10% of increment in the TI of paper compared to the same dose (1 wt%) of unmodified BC. Other authors also studied the cationization of CNC by reaction with diethylenetriamine pentaacetic acid (DTPA) followed by cross-linking with chitosan [77]. The cationic CNC were homogeneously dispersed in water and did not aggregate. The addition of 2 wt% of the cationic CNC increased the TI and burst strength of old corrugated containerboard (OCC) by 40.3% and 46%, respectively. Furthermore, the gloss increased notably and the roughness decreased. Tear strength was slightly decreased.

Korhonen and Laine [63] proved that cationic CNF, produced by reaction with glycidyl trialkylammoniumchloride (GTMAC), could flocculate kaolin particles as fast as synthetic polymers and the system was able to reflocculate. They tried different charge densities and dosages. At the same dose, the flocculation of kaolin increased with the charge density of the cationic CNF, but when the dose was over the optimal, charge reversal decreased flocculation. Experiments were carried out with a thermomechanical pulp containing 40% of kaolin and the retention of kaolin could reach values over 90% with a low charge density CNF (0.4 meq/g). Moreover, the enhancement of mechanical properties with cationic CNF could be even higher than those with unmodified CNF [63].

Lu et al. [74] found that CNF modified with GTMAC can induce significantly flocculation of fines to form CNF-fines complex increasing fines retention (from 86.91% without cationic CNF addition to 91.32% for 5 wt% cationic CNC). The addition of 5 wt% cationic CNF with a high charge density (0.61 mmol/g) increased basis weight (76.2 vs. 78.7 g/m^2^) and density (0.218 vs. 0.265 g/m^3^) of paper sheet which was induced by the improved fines retention in the paper structure caused by the addition of cationic CNF. Additionally, the presence of cationic CNF improved the wet-web strength of paper sheet without affecting the dewatering rate. They also demonstrated that the charge density of cationic CNF plays a positive role in improving the wet-web strength properties.

Huang et al. [75] proved that the Young modulus of paper made of softwood pulp increased from 7.1 to 8.3 GPa when the 10% of fibers were replaced by cationic CNF, while the Young modulus obtained with the same percentage of untreated or TEMPO-oxidized CNF was 7.7 GPa. Similar behavior was observed for TI. This was due to the stronger bonding between cationic CNF and fibers, due to electrostatic attractive forces, and to the higher amount of cationic CNF retained. Although the dose of cationic CNF is high, compared to the dose of retention aids, the potential of their use as retention aids enhancing the mechanical properties of paper has been demonstrated.

Diab et al. [16] prepared cationic microfibrillated cellulose (CMFC) by means of reaction with β-chloroethyldiethylamine followed by quaternization with methyl iodide and assessed the efficiency of CMFC, compared to CPAM, on the retention and drainage of different pulps. Although the efficiency of CPAM as retention aid was higher than that for CMFC, the presence of CMFC did not affect the drainage of the pulp significantly and the mechanical properties of the sheet were notably superior, especially in the case of bagasse pulp with a 20 wt% of GCC. In this case, burst index and tear index were around 2.3 kPa m^2^/g and 4 mNm^2^/g, respectively when CPAM was used, while they reached 3.4 kPa/m^2^/g and 5.4 mNm^2^/g with CMFC. Tensile energy index was doubled (from 0.4 to 1 J/g) and Young modulus was also improved (from 3.8 to 4.7 GPa). The poor retention efficiency of CMFC could be due to their low fibrillation and their low charge density which limited its electrostatic interaction with fillers. The lower content of GCC in the paper sheet contributes to the improvement of the mechanical properties, which must be considered to evaluate the effect of CMFC. When the mechanical properties of bagasse paper with CMFC (0.1%) were compared to those of the paper without GCC and CMFC, the improvements in mechanical properties are lower: Burst index did not change significantly, but tear index increased up to around 15%, to 5.4 mN·m^2^/g and tensile energy index increased up to 45% (from 0.67 to 0.98 J/g). GCC retention and bagasse paper tear index were further improved when CMFC was combined with bentonite forming a dual retention system.

Liu et al. [76] found that 0.4% cationic CNF increased PCC filler and tobacco pulp retention by 31.8% and 81.6%, respectively. Drainage of tobacco pulp was also improved. Bulk and air permeability of the sheets increased by 6.8% and 41.8% and tensile strength was slightly deteriorated.

Ondaral et al. [73] showed that 5 wt% cationic CNF increased the TI value of a softwood Kraft pulp by 50%, but it was up to 60% when cationic CNF was used in combination with TEMPO-oxidized CNF. They observed that the cationic modification of cellulose was less efficient in promoting fibrillation than the TEMPO-mediated oxidation. Thus, the formation of a slightly amphoteric structure after cationization can decrease repulsion forces between fibrils [82]. This research does not show the effect of cationic CNF and the combination of both CNFs on drainage and retention, but the results suggest that the cationization of one portion of CNF could be a method to improve CNF retention in the sheet while improving mechanical properties.

Another interesting approach about the use of cationic CNF is the positive effect on decoupling filler content and sheet strength. Li et al. [83] prepared cationic CNF by means of cellulose etherification with CHPTAC in a NaOH/urea aqueous solution and modified GCC. They observed that the CNF precipitated on the surface of the GCC particles and this improved their retention within the fiber network and the way it interacts with fibers. Consequently, the tensile, tear, and burst indexes improved.

Brockman and Hubbe [68] cationized partly the CNC surface with poly-(diallyldimethylammonium chloride) (poly-DADMAC), a highly charged cationic polymer. The modified CNC strongly promoted water release of the pulp containing PCC and reduced the required amount of poly-DADMAC, as the final additive, by ten times than that of the dual retention system consisting of poly-DADMAC and anionic-PAM.

Another authors focused their research in the increment of the negative surface charge of the NC to increase the hydrogen bonding of NC with the fibers. This contributes to enhance the performance of NC and reduces the required dose, limiting their effect on drainage. The increase of the anionicity of NC is carried out by means of introducing carboxymethyl, carboxylic, or aldehyde groups in the NC structure. When NC are used as coating, modifying their charge to cationic or strong anionic does not affect their efficiency as strength aids as it has been proved by Syverud and Stenius [84]. However, many studies support the highly improvement in mechanical properties reached by the mass addition of modified NC to different pulps.

The carboxymethylation increases the charge of the CNF by the introduction of carboxymethyl groups in the hydroxyl groups of cellulose, affecting their interactions with the fibers. The use of carboxymethylated-CNF can result in a better improvement of the mechanical properties of the sheet, i.e., internal cohesion (z-tensile) and tensile strength, minimizing their effect on drainage [51]. Carboxymethylated-CNF could form a thin layer on the cationic agent adsorbed on the fibers surface instead of blocking pores. This would explain the high drainage rate reached, which is similar to that without CNF [51]. Lourenço et al. 2019 [80] studied the effect of the carboxymethylation pretreatment on the efficiency of CNF on filler retention and tensile strength improvement by comparing with that for TEMPO-mediated oxidation CNF. Carboxymethylation increased the flocculation efficiency of CNF with respect to TEMPO mediated oxidation. However, the reached PCC filler retention was similar in both cases, reaching filler retention improvements around the 90% over that reached without any CNF or additive. Furthermore, the improvement on tensile strength (normalized for the filler content) reached with TEMPO-CNF was higher than that for carboxymethylated ones. The effect on paper sheets containing GCC was not studied.

Cha et al. [79] determined the potential of using carboxylated-CNC to improve paper strength. Carboxylation consists of converting some hydroxyl groups of cellulose into carboxylic groups by means of TEMPO-mediated oxidation of the CNC, increasing the anionic charge density of CNC. The addition of a low dosage of carboxylated-CNC, 0.7 wt%, improved notably the mechanical properties of paper by 24.9% and 34.3% in tear index and TI, respectively. These results are not significantly higher than those reached by Sun et al. [15] for bleached *Eucalyptus* kraft pulp reinforced with the same dose of unmodified CNC. However, Xu et al. [85] proved that carboxylated-CNC allows the improvement of retention and drainage processes if it is combined with CPAM. Xu et al. [85] observed that the carboxylation of CNC by means of TEMPO-mediated oxidation decreased their agglomeration and enhanced their dispersion in the deinked pulp, which intensified their detrimental effect on drainage rate and first pass filler retention, in absence of CPAM. However, the combination of the carboxylated-CNC with CPAM reversed the trends: The drainage rate and first pass retention increased with the charge density of the modified CNC over values reached when CPAM was used without CNC. The use of a dose of 0.8 wt% of the most anionic CNC (degree of oxidation of 0.134) increased the drainage rate by 12%.

Sun et al. [15] modified the CNC by means of a periodate oxidation to introduce aldehyde groups on the CNC surface. This increased their efficiency as a wet-strength additive by means of the reaction between the aldehyde groups and the hydroxyl groups of cellulosic fibers forming waterproof covalent links.

Recently, Campano et al. [17] have demonstrated the potential of using cationic hairy nanocellulose (CNCC) as retention additive. The addition of 20 mg/g of CNCC reduced the pulp drainage time by 78% and improved the filler retention by 77% compared to the recycled pulp without CNCC, without detrimental effects on mechanical properties of the recycled paper. The tensile and tear indexes of the sheets with 20 mg/g of CNCC (40.5 ± 0.8 Nm/g and 6.6 ± 0.3 mN·m^2^/g, respectively) were similar to those obtained without CNCC (41 ± 1.5 Nm/g and 6.9 ± 0.3 mN·m^2^/g).

#### 2.1.4. Dispersion of Nanocelluloses

Among all NC, CNF have shown the highest potential to strengthen paper, mainly due to their high aspect ratio. However, they tend to form hydrogels by hydrogen bonding between the nanofibrils, quite stable and strong enough to hinder their homogeneous dispersion and difficult to mix within the furnish. In this way, some fibrils clusters are present even despite the fact that they apparently seem to be a homogeneous suspension, so their reinforcing efficiency is still less than the one expected [86,87]. Moreover, these clusters decrease the light scattering coefficient and thus opacity of the sheet. In fact, while Gonzalez et al. [46] did not obtain any significant change in the opacity values of sheets after the addition of 9 wt% CNF, disintegrated at 180,000 revolutions, Petroudy et al. [58] reported a significant reduction of this parameter, with 5 wt% CNF disintegrated at 20,000 revolutions. Since the interactions among nanofibers depend on the concentration of the gel, diluted CNF suspension could meet this challenge. However, producing diluted CNF increases transport costs and diluting the gel on site before using it does not guarantee saving shearing energy.

On the other side, CNC suspensions have no gel properties due to their rod-shape morphology with low aspect ratio, thus having a viscosity similar to water. However, the CNC have also a strong tendency to form aggregates such as clusters by hydrogen bonding [31]. When sulfuric acid is used to hydrolyze cellulose, the surface hydroxyl groups of cellulose react to yield charged sulfate esters. They usually promote dispersion of CNC in water, but the substitution degree is not high enough to keep stable suspensions for a long time. Thus, they tend to flocculate and sediment. This effect is even more pronounced when either hydrochloric or hydrobromic acid are used for CNC production [88].

Campano et al. [89] studied different pulping conditions, as well as the use of dispersing agents to assess the best conditions for CNC and CNF mixing in a deinked pulp. The highest increment in TI, (30%), was reached when the recovered paper was soaked before its disintegration, the pulping time was long (60 min), and CPAM was used as retention agent. Alternatively, the use of CNF with a low dosage of dispersing agent (0.003%) got an increment in TI of 20.6%. The dispersing agent was a moisturizing agent, which facilitates the intimate contact between coatings and paper in papermaking industries. Although this method did not maximize the TI, it reduced the energy requirements related to NC mixing. However, the dispersing agents can interact with the coagulants and flocculants used in the wet-end decreasing their efficiency, so a deeper study is still needed.

**Figure 1:**
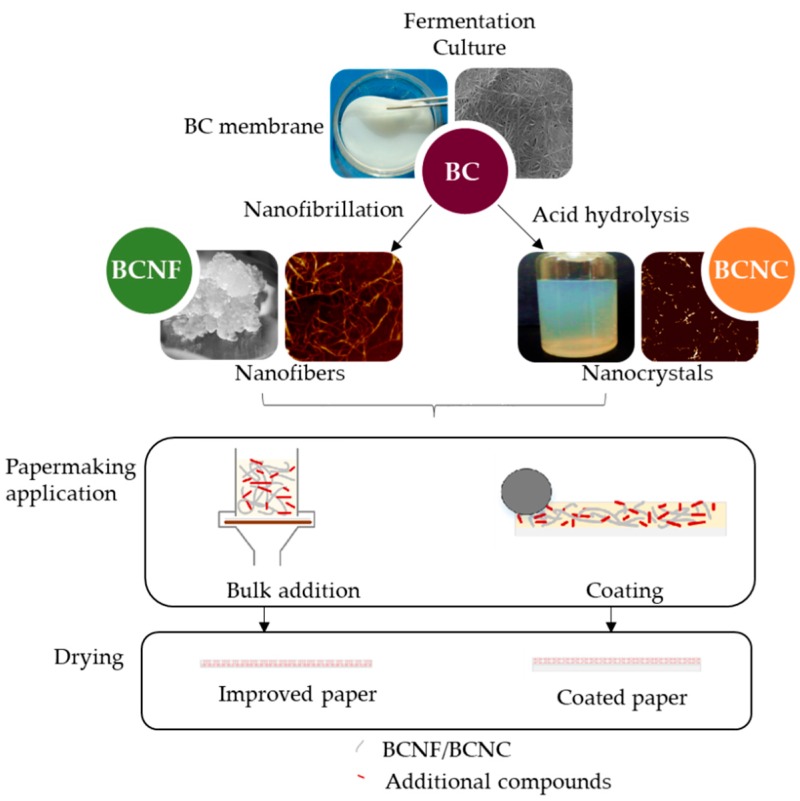
Alternatives for BC dispersion (BCNF: BC nanofibers; BCNC: BC nanocrystals).

Johnson and Winslow [90] were the first in proposing the use of BC as a paper strengthening additive. Xiang et al. [91] and Yuan et al. [92] proved that getting a homogeneous distribution of BC within the paper matrix is a key for successfully reinforcing paper. Most of the researches about that disperse the BC network in water before using it by means of intense stirring [93,94] or acid hydrolysis, as shown in Figure 1 [95]. However, the mechanical defibration of BC is difficult to be implemented in papermaking industries due to the high energy required because of the high entanglement of the nanofibers reached during culture [93]. Chen et al. [37] dispersed the BC in water to get a gel with a concentration of 2g/L after 6 min of blending. However, the blades of the blender cut the fibers affecting the morphology and polymerization degree of the BC. Gao et al. [93] studied the stirring time required to disperse wet membranes of BC in water by means of a high-speed dispersion machine running at 11,000 rpm. They prepared sheets containing different doses of the dispersed BC suspensions and determined their mechanical and physical properties. They found that the most efficient BC suspension was stirred a BC concentration of 0.3 wt% during 3 min. However, they do not clarify if the suspension was added in the disintegrator with the pulp sheets or after disintegration. However, there are other ways to disperse BC in water before adding it to the pulp (Figure 1): (i) Mechanically, by means of nanofibrillation, for example by using a pressure homogenizer, to obtain CNF suspension from BC (BCNF) and (ii) by acid hydrolysis to form a suspension of bacterial nanocrystals (BCNC). The energy requirements of nanofibrillation are high, but the acid hydrolysis requires the use of acid and removes the amorphous part of the cellulose thus reducing the yield.

Recently, Campano et al. [38] used soft homogenization to disperse BC in water forming a gel of nanofibers containing clusters of BC and they added different proportions to a deinked pulp. Interestingly, the remaining of non-dispersed clusters of BC provide flexibility to paper, improving at the same time tensile and tear strengths, as well as strain at break by 11.1%, 7.6%, and 66.8%, respectively, with the addition of 3 wt% BCNF.

### 2.2. Surface Application of the Nanocelluloses

**Figure 2:**
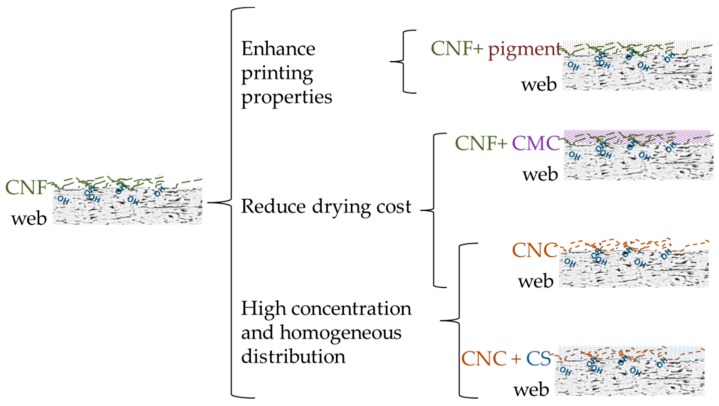
Some strategies in NC surface application (CMC: Carboxymethyl cellulose; CS: Cationic starch).

A strategy to avoid the mixing energy requirements, assure the NC retention, and reduce their effect on drainage is the use of a different application technique of NC, such as the paper coating before drying. With this method, the different components are placed in well-defined layers, thus ensuring an improved paper quality [96] for printing purposes and barrier properties. However, the CNF are easily delaminated at a critical printing speed if they do not penetrate deep enough in the sheet, which is related with its negative charge, the same as macroscopic fibers [45]. Some strategies to avoid delamination, improve drying and homogeneity of coating is shown in Figure 2.

In this sense, the use of pigments such as clay or calcium carbonate, improves the CNF distribution on the surface of the wet-web, improving notably the physical and printing properties [40,97,98]. For instance, Ridgway and Gane [99] retained CNF on the surface of a copy paper by the application of a previous porous coating layer of modified calcium carbonate. With this approach, stiffness was increased by 66.7%.

The CNF can be combined with active functional aids to give special properties to the paper or board surface, for example antimicrobial, antioxidant, aromatic, flame retardant, electric, or catalytic properties [100,101,102,103]. Although the active aid can bind the fiber surface, the higher specific surface of CNF enables to obtain a paper surface with a higher concentration of active aid while limiting the amount of active aid required. Furthermore, the active aid can be fixed on CNF or CNC and deposited in the paper surface by printing. This has many potential applications, such as microfluidic paper devices, chemical compounds delivery, smart packaging or traceability, etc. [104].

The concentration of CNF is limited by the viscosity of the CNF gel, being highly viscous even at concentrations below 1 wt% [43]. However, the CNF viscosity is reduced with the shear rate. With the use of spray coating, the formation of a very thin and homogeneous layer on the surface of the sheet can be produced, reducing the CNF consumption, but a very dilute suspension is required, the fact that increases the energy spent in the drying section. Brodin et al. [6] compared the different CNF coating strategies and they concluded that the most important point is the use of high shear rate during the CNF dosage, due to the thixotropic character of CNF.

With the use of some additives, the rheology of CNF suspensions can be modified, thus easing the distribution on the paper surface. In this context, Mousavi et al. [44] mixed CNF and carboxymethyl cellulose (CMC), which acted as dispersant, allowing a higher CNF concentration in suspension and producing more homogeneous coating. As a consequence, the mechanical and barrier properties of coated paperboards were improved considerably, while the reduced water content of the coating decreased the drying cost.

The suspensions of CNC are easier to manage for a coating application as they have a lower viscosity than the CNF gel. As a result, CNC have been also applied on surface-sized paper to improve its mechanical properties and resistance to air permeability [105]. Yang et al. [105] combined CNC with cationic starch (CS) at different proportions, and applied it as a coating on paper. The use of CNC in coating formulations improved the mechanical and barrier properties of CS coatings, which, otherwise, would not fill the requirements of packaging materials. The optimal CNC percentage in the CNC/CS mixture was as low as 0.3 wt% respect to dry CS. The use of these coating formulations reached increments of 6%, 9%, 23%, and 4% in tensile index, tear index, folding endurance, and burst index, respectively, compared to those of the paper covered by CS.

#### 2.2.1. Barrier Properties

To ensure a good food preservation and avoid the transmittance of non-desired components present in the atmosphere, the oxygen transmission rate (OTR) of the food packaging must be under 20 mL/m^2^·day·atm [106]. According to the literature, paper coated with CNF can decrease the OTR to 3 mL/m^2^·day·atm, being even lower than that for many petroleum based polymers, such as polyethylene [106,107]. This enhancement in barrier properties is due to the reduction of paper porosity that forces the oxygen molecules to pass through a long and highly tortuous path to reach the other side of the network.

Moreover, with the use of CNC, the OTR can be reduced even below 1 mL/m^2^·day·atm since the size of CNC is lower than CNF [108]. Moreover, the high interaction of NC with water is mainly due to the amorphous parts of the cellulose, so a lower sensitivity to moisture could be expected if CNC are used instead of CNF. Furthermore, during cellulose acid hydrolysis with sulfuric acid to obtain CNC, some of the hydroxyl groups of the surface chemistry of the particles are substituted by sulfate ester groups, thus decreasing the interaction with water and, consequently, the OTR can be reduced. Rampazzo et al. [108] used CNC to coat poly (ethylene terephthalate) films in order to reduce their gas permeability. Oxygen and carbon dioxide permeability values measured at 0% relative humidity (RH) were hundred times lower than those of equal thickness of common barrier synthetic polymers, over a broad range of temperatures. However, they observed a fast but reversible increment in the gas permeability with the increase of the RH.

The OTR increases with the presence of atmospheric humidity due to the extremely high swelling ability of NC, thus water molecules are retained in the NC network and oxygen can diffuse more easily through adsorbed water increasing the oxygen permeability and decreasing the barrier properties of the NC coatings [101]. Water vapor permeability of papers made with a proportion of NC is high, i.e., one order of magnitude higher than that for polyethylene [47]. The temperature and RH of the air were found as the most influencing variables in water vapor permeability of paper: It rapidly increases when RH is higher than 25% and it follows the Arrhenius law for temperature [109]. In food packaging RH is typically higher than 60% which limits the use of NC. Therefore, protecting NC coating against water is a main challenge of their use in packaging. This can be achieved by means of using crystalline minerals, by hydrophobic coatings or by functionalizing NC.

Several authors have successfully used crystalline minerals such as montmorillonite or other clays in combination with CNF to improve the barrier properties in presence of high RH environments [42,110] due to crystalline minerals that are completely impermeable to water, gas and oil, because of the increase in the tortuosity of the path for them.

On the other hand, the combination of NC with hydrophobic coatings can protect the paper from the water and water vapor, and keep the barrier properties at high RH. Hul, et al. [111] used shellac as hydrophobic coating to protect the CNF layer. Shellac is a natural, biodegradable, and non-toxic resin secreted by the lac insect *Kerria lacca*. Although shellac has no gas barrier properties, it reduced OTR in one magnitude order because it covered the pores of the CNF layer. Aulin et al. [112] used polyethyleneimine (PEI) with the same aim. They used a multilayer coating consisting of intercalated layers of PEI and CNF or PEI and CMC on a PLA substrate. Since CNF are anionic, the cationic charge of PEI improves the interaction between the layers. However, PEI does not accomplish biodegradable and safety standards expected from the biodegradable packaging. Ankerfors et al. [113] studied the multilayering of a chemithermo-mechanical pulp (CTMP) using cationic CNF/anionic MFC, cationic polyamideamine epichlorohydrine resin/anionic CNF, and cationic starch/anionic starch. They got an increase in the wet strength of the paper, but barrier properties were not studied.

The functionalization of NC is another way to modify the OTR. At 0% RH, carboxymethylated CNF reduced the OTR by three orders of magnitude compared to unmodified CNF, but it was only preserved up to RH below 30% [47]. The increase of hydrophobicity of NC surface can reduce the hydrogen bonding ability of NC with water, improving barrier properties, and wet strength. However, the density of the network could decrease because it depends on the number of hydrogen bonds between nanofibrils, which could be reduced as a consequence of the chemical modification of NC surface. Hydrophobization can be reached without using chemicals, by means of controlled hornification, since it decreases the swelling ability of CNF. Therefore, it improves the gas and water vapor barrier properties in presence of moisture. Sharma et al. [114] applied a successful treatment for CNF films hydrophobization consisting of heating the CNF up to 175 °C for 3 h. The OTR of the films decreased from 0.18 to 0.01 mL·µm/m^2^·day·kPa and their water vapor permeability decreased from 55 to 27 g·µm/m^2^·day·kPa. However, it increases the cost, fact that may be unfeasible for papermaking and packaging industries.

In an attempt to improve wet-end additives and improve paper properties, Yuan et al. [115] added BC to an alkyl ketene dimer (AKD) sizing agent. They optimized the dosage of BC, the retention system, and the sizing enhancement agents. The addition of BC had a negative effect on the AKD sizing in absence of the retention system because of the poor retention of BC. However, sizing was notably improved (up to 60%) when either 0.5% of CS or 0.02% of cPAM were used. Both CS and PAM are cationic polymers that interact with anionic groups of cellulose, improving the BC retention. On the other hand, they do not improve AKD sizing in absence of BC, which demonstrate that the enhancement is caused by the BC.

Furthermore, the low permeability of NC to oils and greases is due to the high amount of hydrogen bonding, strength, and compactness of the NC network. Due to that it keeps a close relationship with the permeability of non-polar gases like oxygen [42]. Interestingly, some of the published studies about the oleophobization of films showed superior barrier properties for oxygen permeation [116]. The tried methods in those works consisted of modifying NC with low surface energy substances, such as fluorocarbons. In addition, a higher effectivity can be achieved when the surface of NC layer is pretreated with nanoparticles prior to treatment with perfluorosilane, since it decreases the roughness of the surface. Kisonen et al. [117] reached oil-impenetrable films composed of CNF and O-acetyl-galactoglucomannan (GGM). Both low and high substituted GGM avoided the grease permeability, even at high temperatures, obtaining different water repellence. In addition, these coatings improved the excellent oxygen permeability and stiffness reached by the CNF, even at high RH, up to 90%.

Moreover, the OTR, water vapor permeability, and oil-permeability of the NC and its dependence with the RH are also influenced by the impurities present in the NC such as lignin content. The presence of lignin in the NC increases their hydrophobic character, keeping good barrier properties even in presence of humidity. However, Spence et al. [118] showed that water vapor permeability increased in presence of lignin because of the weaker interactions among fibrils, which reduces the compactness of the network.

#### 2.2.2. Alignment of the Nanocelluloses in the Paper Matrix

Recently, different studies support the idea that the alignment of CNF and CNC can improve both the stiffness and the strength of NC films [119,120]. Moreover, in the papermaking industry, cellulose fibers are mostly lengthwise orientated, to get a dimensional stability of individual fibers in their length-wise dimension, even when subjected to large changes in moisture content [2]. In this context, a better orientation of either CNF or CNC may lead to a greater increment in mechanical properties, as well as a better dispersion of the individual nanoparticles. Different attempts have been tried to orientate these NC in the last decade [121]. One of the most common methods to orientate highly concentrated aqueous CNF gels is wet stretching [122]. This process can be used to coat paper, since it generates high in-plane orientation, reaching order parameters between 0.6–0.8, where 0 is random orientation and 1 perfect alignment. However, the high cohesion necessary to drive sustaining direct and fast stretching procedures is a bottleneck to progress with this method.

The use of fluid dynamics to align the CNF and CNC has been presented as a common method to produce NC filaments with high strength of nearly 500 MPa with extensional flows at low shear [123]. Moreover, external magnetic fields [124] or electric fields [125] have been also used to align CNC. Li et al. [126] fabricated unidirectional reinforced nanocomposite paper with CNC and wood pulp as a matrix under an externally applied magnetic field. They showed that, compared with control paper sheets made from wood pulp, the storage modulus increased dramatically from 652 MPa to 4.88 GPa in all the cellulose nanocomposites prepared. Nevertheless, the required magnetic flux density to align the CNC was quite high (7–10 T) due to the small magnetic anisotropy of CNC. It is also worth mentioning that despite the fact that high magnetic flux densities are able to orient the liquid crystal helices of CNC with respect to the field, the magnetic torque is so small that the helical arrangement cannot be unwinded [121]. Nystrom et al. [127] aligned CNC in wrinkled polydimethylsiloxane templates, transferring them to polyethyleneimine (PEI)-coated silica surfaces with a printing process similar to microcontact printing. They created an advanced multilayering method, by repeating the transfer process at a 90 degree angle, creating a network structure. By the application of this method to the surface coating of paper, different benefits, such as improved strength in a specific direction, can be obtained in a controlled way. The alignment of modified CNF or CNC can be also used to produce high special papers with specific new directional properties.

## 3. Drawbacks in Methodology to Characterize Nanocelluloses at Industrial Scale

In general terms, one of the main drawbacks for nanomaterials full-scale production is the lack of fast and robust characterisation methods to control their production and quality along the production chain. Nowadays, there are few measuring tools capable to characterize NC at the industrial plants getting reliable, fast, and accurate measurements in a cost-effective way.

In academic studies several methods have been used to determine the size, size distribution, shape, mechanical properties, crystallinity, nanofibrillation degree, water retention value, etc. of NC suspensions [128,129,130] but, for the moment, no systematic and streamlined methods exist for large-scale quality control characterization in pilot plant production and beyond [131]. Furthermore, there are no measuring standards for NC products, nor regulation or safety guidelines.

NC properties, including their dispersion and stability over time, are critical in several industrial unit operations, such as pumping, mixing, storage, application, and filtering, as well as for the quality improvement of the final products. Considering that not all NC ventures have been successful and the high investment required for full commercialization, the gap of NC quality control during its production and application must be filled in to develop sound value propositions.

### 3.1. Dimensions and Structure

For spherical nanoparticles there are robust protocols to determine their size and shape using a range of particle counting and ensemble methods. However, for more complex nanomaterials such as NC, the measurement of their shape or network and size distribution is considerably more challenging than for well-behaved spherical nanoparticles with monomodal size distributions since they have an irregular rod-shaped structure for CNC or entangled network for CNF, broad size distributions, and a strong tendency to aggregate or agglomerate. Therefore, an important impediment to commercial progress with NC is to find characterization techniques that can rapidly determine the dimensions of NC in the dispersion state considering the difficulty to have stable suspensions with minimal aggregation or agglomeration and the large amount of potential NC samples coming from different cellulose resources and production methods.

Kaushik et al. [131] reviewed the transmission electron microscopy (TEM) technique to obtain high quality images of CNC analyzing the sample preparation and the use of different contrast enhancement techniques (e.g., negative staining, metal shadowing). They concluded that an automated particle measurement procedure using TEM image analysis software is required to streamline quality control. Jakubek et al. [132] have optimized the methods for depositing CNC on solid supports for atomic force microscopy (AFM) to minimize particle agglomeration while maximizing the number of individual particles per image. They have also evaluated the effects of sonication on the particle size distribution and the effects of analyst bias on image analysis by AFM.

The AFM, TEM, and scanning electron microscopy (SEM) are time consuming, because they require preparing the sample on specific supports and analyzing a high enough number of images to have a representative size distribution. In addition, sometimes the desired information is not found. Several authors have demonstrated the usefulness of scattering techniques to determine the dimensions of dispersed NC. Statistically averaged cross section dimensions of CNC and CNF can be determined by means of small-angle neutron scattering and small-angle X-ray scattering [133] by modeling them as rigid parallelepipeds with very high aspect ratio as the length of nanofibrils is much larger than their section. The use of dynamic light scattering (DLS) for dimensional characterization of NC presents several challenges: First, it requires dust-free sample preparation because microparticles of dust movement is much slower than that for nanocellulosic particles and they can saturate the detector resulting in a false measurement result. Second, the length of NC particles usually reaches the submicron or even micron scale which makes both DLS measurement and data interpretation challenging [133]. Finally, DLS gives the hydrodynamic dimensions assuming that particles are spheres and the particle size is determined from that considering that the particle concentration is low enough to assure that diffusion takes place only by means of Brownian movement and the aggregation of particles is impaired; particle flexibility and surface charge can interfere the measurement too [134]. 

There are methods to characterize the NC length based on indirect measurements, as the measurement of the gel point, from which the aspect ratio can be calculated [135,136,137]. The gel point is calculated from the sedimentation curve of the suspension as the volume concentration in the boundary between dilute and semi-dilute region [137,138]. The gel point has been used with macroscopic fibres and microfibrillated cellulose, that easily sediment by gravity [136,139,140]. This methodology has been recently modified to be applied also with high fibrillated NC by Sanchez-Salvado et al. [141]. In this case nanofibers are dyed and longer times are required.

Another important parameter is NC dispersion. Some researchers have studied the effect of the NC mixing with the pulps on the mechanical paper properties [89] or the use of dispersant agents to improve the NC dispersion in a polymeric matrix [87,137,140,142]. However, there is no methodology to quantify the dispersion of the NC hydrogels in a water suspension before its application yet, although it may have a big influence on NC.

### 3.2. Retention

Ankerfor, et al. [143] studied the quantification of non-retained NC in an unbeaten pulp, by determining the concentration of sugars of the filtrate. However, a complex pulp suspension, such as deinked pulps, not only fibers are present, but also fillers, colloidal material, and fines, which usually passed through the wire. In addition, NC is typically used in low dosages. Thus, a direct quantification of their retention is still needed. Usually NC retention has been indirectly associated to its effect on the physical and/or mechanical properties of paper, such as porosity or tensile strength [41].

### 3.3. Rheology Behavior

Several important challenges in the application of NC hydrogels in papermaking such as their mixture with pulp or their spreading on the web surface, and in other uses as 3D printing, food industry, etc. are related to their high viscosity or their complex rheology. Therefore, there are many studies on rheological behavior of these NC hydrogels, although many of the studies deal with the effect of NC on the rheology of polymers or other materials [144].

The rheology behavior of NC hydrogels depends on the interactions among the nanofibrils and between them and water. Therefore, it is determined by the dispersion grade, which depends on the concentration, morphology and surface properties of NC [145]. The dispersion grade is determinant for rheological behavior, since the NC suspensions can have two different kinds of behaviors: Suspensions with concentration over the critical one and intact network, being hydrogels showing a plastic behavior; but suspensions with concentration below the critical one or after network disruption, due to shearing forces, can even behave as Newtonian fluids depending on the concentration and shearing history [146,147].

The close relationship between the morphology, nanofibrillation grade, and viscosity of the hydrogels has caused that several authors have proposed the study of the rheology behavior as a way to characterize NC [148,149]. Hubbe et al. [144] have reviewed the main studies published on this topic and deeply compared the different results. They compiled all the viscosity results for CNC, CNF, and BC suspensions and observed that viscosity increased with solids concentration with different dependence in function on the NC morphology. The viscosity of CNF was more dependent from solids concentration than CNC for suspensions below 1 wt%. However, the viscosity of some CNC suspensions was more dependent from solids concentration than others. They explained that the viscosity of CNC suspensions increased with the aspect ratio and that some conditions (pH, ionic strength) could favor the formation of structures with higher viscosity, such as gel-like structures.

The main challenges in rheological measurements of NC suspensions have been already summarized by Hubbe et al. [144]:

1. Wall slip at laminar shearing because of the strong interaction of fibrils or CNC that separated from the measure devices surfaces, especially if those are smooth, creating a lubricating water layer next to the surface while the most part of the NC suspension is not flowing. This challenge is common to all the cellulosic suspensions and it was first observed with pulps [138,150,151].
2. Shear banding when laminar shearing start breaking by Taylor vortices. Then, the fibrils or CNC are moved to some of the regions in the suspension creating zones with a higher solids concentration than others. It could be expected that in CNF hydrogels with high network strength this effect could be minor. However, the fact is that the CNF hydrogels are susceptible to suffer this shear bonding since the network has always some weaker points and they show a thixotropic behavior. Shearing breaks the network structure forming flocs with free water between flocs. The rheology of the suspension changes with the evolution of the hydrogel from the viscous solid behavior to a discrete flocs suspension behavior as the network disruption occurs. This explains shear thinning of NC suspensions.
3. Fibrils alignment can occur if low shearing is kept during a long enough time giving rheopectic behavior in these cases.
4. Combination of reversible effects such as the elastic behavior of hydrogel and irreversible effects, such as the disruption of fibrils network.

Nechyporchuk et al. [152] studied deeply the wall-slip and shear banding in CNF hydrogels looking for a solution. They concluded that the use of rough tools or the attaching of sandpaper to the smooth ones, prevents these in the case of TEMPO-mediated oxidation CNF hydrogels, but not in the case of enzymatic CNF.

The influence of the dispersion and the swelling of the CNF suspensions was deeply studied by Šebenik et al. [153] who found at the rheological measurements a way to determine the dispersion grade of the TEMPO-mediated oxidation CNF suspension when they were prepared from freeze-dried powder. Although the powder was dispersed in water by combining shearing and sonication, a maturation of several days was required to get the formation of an homogeneous completely swollen network structure since the zero shear viscosity of the suspensions increased with the time, following an sigmoidal function, during days. However, in the case of highly concentrated CNF, solids concentration around 4%, the use of rough plate-plate geometry could increase the shear banding forming lumps that increase the measurement error over the affordable [154]. This is relevant for 3D printing studies, where high consistency is required for the hydrogels. In these cases, the measurement can be conducted by applying an oscillatory stress, to a piece of gel, with a fixed oscillation frequency at a rheometer. With this kind of device, Kyle et al. [155] compared the rheology of CNF, CNC, and CNF-CNC blends suspensions and they found that blending CNF and CNC increased the viscosity and stiffness of the hydrogel requiring a higher shear rate to induce the fibril alignment. This probes that the presence of CNC in a CNF hydrogel strengthens the network.

## 4. Market Perspectives

Despite the excellent properties of NC, confirmed through two decades of intensive research, the NC market is still low (2500 [156]–10,000 [157] ton/year). There is a high variety of NC with very different characteristics and costs depending on the production method and conditions [10,26,28,30]. Due to this, the prospective for potential market highly varied among the different published data. For example, recent reports published [158] predicts that the NC market is expected to increase at a compound annual growth rate superior to 18% during the forecast period, 2019–2023, reaching €576.5 million by 2023, while Biobased Markets estimate a higher growth up to 30% achieving around 250,000 metric tons in 2025 [157,159]. NC is mostly used in paper, board and packaging (36% of the demand), composites (25%), and filtration products (19%) [156]. Shatkin et al. [160] in 2014 estimated the worldwide uses for NC in several sectors considering the overall production of each sector. They projected the largest worldwide uses for NC to be in packaging sector (11.8 million metric tons), paper and board industry (4.56 million metric tons), automotive components (4.16 million metric tons), cement (4.13 million metric tons), plastic film replacement (3.37 million metric tons), hygiene and absorbent products (3.24 million metric tons), and textile for clothing (2.54 million metric tons) [160]. The total annual consumption of NC for low-volume applications was estimated to be 2.23 million metric tons, which would represent less than 10% of the high-volume applications [160]. Chauve and Bras [161] analyzed the marketing challenges for NC and elaborated a strength, weakness, opportunities, and threads matrix for CNC and CNF. The higher complexity and cost and lower yield of CNC production compared with those for CNF were some of the weakness of the CNC; but the potential of production cost optimization was higher for CNC. In that time there was no commercial success evidence for CNC, and it was poor for CNF [161].

Twenty years ago, the industrialization of NC was limited to a few companies and research centers, mainly from Scandinavian countries, France, and Japan in case of CNF production and North American for CNC. Nowadays, key producers in the NC market include FiberLean Tecnologies (UK), Kruger (Canada), Borregaard (Norway), CelluForce (Canada), GranBio (USA), Melodea Ltd., Chuetsu Pulp and Paper (Japan), Nippon Paper Industries Co. Ltd. (Japan), and CelluComp (UK), etc. FiberLean, Kruger, and Borregard are the largest producers of CMF with 8800, 6000, and 1000 metric tons per year, respectively. CelluForce operates the world’s largest CNC plant which is capable of producing 300 metric tons per year [162].

A study on NC in packaging [159] estimates that 30,000 additional NC tons were produced in 2018 in paper mills and used on site in their own products (mill captive), which represents a 75% of the total NC production [159]. This saves NC drying, redispersion, or transportation cost and allows producing the NC with the minimum quality required for its specific application and online controlling of the NC properties and production on time to adjust them to the production needs. The CNF gel concentration can be selected to avoid challenges related to homogeneous dispersion of CNF in the pulp. Any cellulosic stream can be used as raw material, even waste streams, with the benefit of reducing waste generation [163]. However, the quality and reliability of CMF and CNC cannot be properly ensured for extensive industrial use, since there are no technologies capable of providing their fast characterization in industrial environments [130]. Current nano-characterization methods are based on offline measurements performed in laboratory facilities that require complex and time-consuming post-processing and analysis, as well as involve high capital investment and highly qualified personnel.

New advanced materials such as NC take a long time to merge and to be accepted by both industries and customers. Nowadays, most applications of NC have not grown enough to provide funding for a commercial scale investment, and, therefore, the industry is moving across the investment gap also called the Valley of Death, and finding funding where it is needed. For paper and board industry, the challenge of developing the value proposition is almost solved with in situ production [159]. Recent researches have demonstrated that the use of CNF isolated from bleached virgin fibers is not necessary to enhance many recycled paper products [164,165]. The addition of 3 wt% of CNF produced from recycled cellulose streams increased tensile index of recycled ONP and OCC by 30% and 60%, respectively [164]. A recent report from Biobased Markets estimated that more than 75% of all NC in 2018 was produced by paper and board mills and used on site to enhance their own products [159]. Most of these data and industrial applications of NC is unreported for confidentiality reasons but, it is a fact that nowadays several paper mills such as Stora Enso, Norske Skog, BillerudKornäs, or International Paper are studying and working with the use of NC in their facilities [157]. Kajanto and Kosonen [166] have published the results from the use of CNF, produced at UPM’s pilot facilities, on a high-speed pilot paper machine using pulp and process waters from the mill. Trials were done with 1–2 wt% CNF mixed with machine-chest chemical pulp with no fillers, which contained a small amount of wet-end starch, on a narrow pilot paper machine capable of high operating speeds up to 900 m/min. Results showed good runnability of the paper machine, no web-breaks, and good paper formation. The final paper had higher tensile strength, enabling up to 8 g/m^2^ grammage reduction, slightly lower scattering coefficient, and 20%–30% lower porosity. Dewatering in wire-section was reduced, decreasing the solid content by 1%, whereas dry matter increased after wet-press section. Hence, new NC applications are emerging as a green substitute for specific markets with high-volume applications (such as paper, construction, nanofilms, paintings, etc.) and high-added value applications (such as biomedicine, automotive, flexible electronics, etc.). As an evidence, the global market for NC is expected to increase from 2500 to almost 35,000 ton/year by 2030 [156].

However, for other large-scale NC applications collaboration between end-users and producers is required to grow faster, to develop the value proposition through the entire supply chain and, consequently, to close the gap [157]. Therefore, several consortium have been created to promote the large-scale industrialization of NC such as Nanocellulose Forum in Japan, Bio-Based Industries Join Undertaking (BBIJU) in Europe, and Alliance for Pulp and Paper Technology Innovation (APPTI) in USA.

In general terms, the main drawbacks to commercial progress of NC are the cost-competitiveness with traditional technology and the availability of volumes relevant for large-scale industrial use. The increase in paper production cost due to the use of CNF depends on its dose and production method. Considering that the dose were 3% on dry weight, the in situ production and use of CNF would cause a paper cost increase from 60 to 300 € per ton of paper, being the TEMPO oxidation pretreatment responsible for the major part of that [7,167]. Therefore, different strategies to reduce the production cost of NC and to promote their implementation at industrial scale have been studied over the past years. During the last decade, different pretreatments such as enzymatic hydrolysis, carboxymetlylation, cationisation, mechanical refining, and TEMPO-mediated oxidation, before to fibrillation, were developed to reduce the energy requirements for mechanical process from values higher than 100 to only 2–4 Kwh/Kg [5,168].

Moreover, since the chemicals are also of high cost and difficult to recovery, the NC prices today are still high and vary from 50 to 1000 €/kg on dry weight, which cannot be tolerated for large-scale products [156]. Some attempts have been carried out to recover and recycle TEMPO oxidation chemicals for improving the environmental impact and cost efficiency of CNF production. The high cost in TEMPO-mediated oxidation reaction are mainly due to the price of TEMPO catalyst which varies from 120 to 170 €/kg depending on its purity and quantity and the price of NaClO prices which is up to 2000 €/ton [169]. Since the 80s, several researches have studied the electrolysis of sodium chloride to produce sodium hypoclorite [170,171]. Recently, Kuutti et al. [169] have demonstrated that electrolysis is a feasible method for hypochlorite recovery using TEMPO oxidation filtrates in which concentration of sodium chloride was the most important variable which affects the hypoclorite conversion. However, some TEMPO catalyst was degraded during electrolysis process and, therefore, they proposed first to recover the TEMPO catalyst by solid phase extraction (SPE) using a combination of hydrophobic resin material and distillation, and then carry out the regeneration of hypochlorite by electrolysis [169]. On the other hand, Patankar and Renneckar [172] reported the synthesis of heterogeneous magnetic TEMPO catalyst which it was recovered from the reaction mixture and reused avoiding the release of the catalyst into wastewater streams during CNF synthesis. At room temperature, the carboxylic content and rate of oxidation with heterogeneous catalyst were 0.54 mmol/g and 0.085 mmol/g∙h, respectively, which were slightly lower than the values obtained by homogeneous TEMPO-mediated oxidation, carboxylic content of 1 mmol/g, and rate of oxidation of 0.48 mmol/g∙h, but the oxidation level was enough to favor the fibrillation and, thus, CNF production.

## 5. Concluding Remarks

The use of NC in papermaking can contribute to overcome the limits of paper products in terms of strength and barrier properties, obtaining special paper products able to compete with other materials. Furthermore, the use of NC in food packaging can provide functionality to biodegradable packages, ability to control microbial population in the food, and the release or capture of specific substances. This requires solving the sensitivity of the barrier properties to moisture. Other special benefits can be supplied by the use of NC, such as linting control, and fire proof properties, for example.

Despite the huge research work carried out on the production process and applications of NC in papermaking, research is still required to get the optimal solution to overcome the challenges of NC production cost and their use in papermaking, especially those related to their dispersion in the pulp and their effect on retention and drainage. The key to decouple the retention of NC and the drainage rate is the optimization of the wet-end. This depends on the interactions between NC, retention systems, and the other components of the pulp. The huge variety of factors involved in the NC performance causes that the achieved solutions are specific for each paper product and process. Therefore, the in situ ad-hoc production of NC with the required properties to optimize the product quality with the minimum cost is one of the most promising ways to reach the economic viability of the use of NC in paper production.

Finally, the use of NC as reinforcement of paper could affect the recyclability of paper as a consequence of the increase in wet strength of the paper or board. This effect should be evaluated since it affects the life cycle of the product.

## Figures and Tables

**Table 1:** Synergies between retention systems and NC (effects on retention and drainage with respect to the pulp with RS but without NC).

NC Type	Pulp	RS	Effect on Retention and Drainage	Increase in TI	Ref.
Carboxylated CNF from ECF Birch Kraft pulp	ECF pine kraft	CS (1.5%)	Drainage rate increased up to 10% (3% CNF)	25% (3% CNF)	[51]
Enzymatic and mechanical CNF from bagasse soda pulp	Bagasse soda pulp	CPAM (0.1%)	Kept drainage rate	30% (1% CNF)	[58]
Enzymatic CNF from BEKP	BEKP (34SR)	CPAM (0.02%)	Preflocculation of PCC with CNF. Filler retention increased up to 3% (3% CNF)	20% (3% CNF)	[61]
TEMPO CNF from BEKP 0.6 and 1.5 mmol/g COOH	BEKP (34SR)	CPAM (0.02%)	Preflocculation of PCC with CNF. Filler retention increased up to 4% (3% CNF of 0.6 mmol/g COOH) Filler retention decreased (3% CNF of 1.5 mmol/g COOH)	Wet strength increased up to 100% at 20% moisture	[57]
Mechanical CNF from semichemical wheat straw pulp	Semichemical wheat straw pulp	CS (0.5%) –colloidal silica (0.8%)	Drainage rate decreases, 105% (3% CNF)	28% (increase in breaking length no TI data) (3% CNF)	[59]
TEMPO CNF from BEKP	DIP	CS (0.5%) –colloidal silica (0.8%)	Decreased drainage rate, 14% higher ºSR (1.5% CNF), but the beating causing similar ºSR got a lower increase in TI	41% (1.5% CNF)	[62]
		Chitosan (1 mg/g)	Increased drainage rate up to 50%	16% (1%–1.5% CNF)	[41]
		CPAM (0.5 mg/g)	Increased drainage rate up to 40% (0.5% CNF)	Decreased by 15% (1.5% CNF)	
		CPAM (0.5 mg/g) – Bentonite (0.5 mg/g)	Increased drainage rate 15% (0.5% CNF)	15% (1.5% CNF)	
TEMPO CNF corn stalk organosolv pulp	DIP	Chitosan (1 mg/g)	Increased drainage rate up to 50% and retention of fillers up to 5%	14% (1%–1.5% CNF)	
		CPAM (0.5 mg/g)	Increased drainage rate up to 40% (0.5% CNF)	Decreased by 5% (1.5% CNF)	
		CPAM (0.5 mg/g) – Bentonite (0.5 mg/g)	Increased drainage rate up to 25% (0.5% CNF)	31% (1.5% CNF)	
Mechanical CNF from bleached organosolv corn stalk pulp	DIP	Polyquaternary ammonium chloride (0.7 mg/g) – CPAM (0.7 mg/g)	Increased drainage rate up to 20% Increased total solids retention	10% (0.5% CNF)	[35]
		Chitosan (2 mg/g)	Kept drainage rate Increased total solids retention	10% (0.5% CNF)	
TEMPO CNF from bleached organosolv rape stalk pulp	DIP	Chitosan (2 mg/g)	Increased drainage rate up to 20%	5% (0.5% CNF)	
TEMPO CNF* from bleached hardwood kraft pulp	TMP + 40% kaolin	PEI (0.2%) – CNF (2%)	Increased retention of filler from 40% up to 95%	No data	[63]
Mechanical CNF from softwood alpha – cellulose	Hardwood pulp + softwood pulp	CS (0.5 and 0.7%)	No data	15%–20% (increase in breaking length no TI data) (3% CNF)	[64]
		CPAM (0.03 and 0.05%)	No data	45%–48% (increase in breaking length no TI data) (3% CNF)	
		CS (0.5%) + CPAM (0.03%)	No data	59% (increase in breaking length no TI data) (3% CNF)	
CNC* with different charge densities	DIP	CPAM – CNC (up to 1%)	Increased first pass retention and drainage rate up to 8%	No data	[65]
CNC* with different lengths	Recycled office white paper	Poly-DADMAC (0.05%) – CPAM (0.05%) – CNC (0.4%)	Increased drainage rate up to 20% Increased retention of fines and fillers (decreased turbidity of filtrate from 50 to 10 NTU)	No data	[66]

BEKP: Bleached *Eucalyptus* Kraft pulp; CS: Cationic starch; CPAM: Cationic polyacrylamide; DIP: Deinked pulp from old newspaper (ONP) and old magazine paper (OMG); DTPA: Diethylenetriamine pentaacetic acid; ECF: Elemental chlorine free bleached; PCC: Precipitated calcium carbonate; PEI: Polyethylenimine; Poly-DADMAC: Poly-(diallyldimethylammonium chloride); TEMPO: (2,2,6,6-Tetramethylpiperidin-1-yl)oxyl; TMP: Thermomechanical pulp. Doses on dry solids.* These NC have been used as anionic microparticles of the retention system.

**Table 2:** Effects of modified NC on papermaking (effects with respect to the situation without NC).

Modification	NC	Agent	Pulp *	Effects on Papermaking	Effects on Paper Properties	Ref
Cationization	CNF	GTMAC	BKSP	No data	Dose of 5% CCNF + 5% ACNF Increments: • TI: 65%	[73]
				Fines retention increased from 87% to 91% (5% CNF)	Dose of 5% (charge density 0.61 mmol/g) Increments: • Basic weight: 3% • Density: 22% • Breaking length: 46% • TEA: 39%	[74]
		Raisacat-reagent	TMP with 40% kaolin	Filler retention of more than 95% (1.5% CNF; charge density 0.41 meq/g) Maximum filler retention of 80% (0.5% CNF; charge density 1.37 meq/g)	No data	[63]
		No data. Surface charge: 0.69 meq/g	Softwood pulp	No data	Dose of 10% Increments: • Young Modulus: 17% • Tensile strength: 25% • Strain: 15% • DC Breakdown strength: 21%	[75]
		β-CEDEA + methyl iodide	Bagasse pulp	GCC retention 15% (1% bentonite) No differences in drainage	Dose of 0.1%. Increments: • Tensile energy index: 42% • Elongation: 42% • Tear index: 13%	[16]
		Etherification	Tobacco pulp	PCC filler retention and pulp retention increased to 32% and 82%, respectively (0.4% CNF) Improved drainage pulp by ≈ 10% (0.4% CNF)	Dose of 0.4%. Increments: • Tensile strength: 10% • Bulk: 7% • Air permeability: 42%	[76]
	CNC	DTPA + chitosan	OCC	No data	Dose of 2%. Increments: • TI: 40% • Burst strength: 46% • Tear index: −5% • Increased gloss	[77]
	CNCC	GT	Recycled deinked newspaper pulp	Filler retention increased by 77% and drainage time reduced by 78% (2% CNCC)	Dose of 2%. Increments: • TI: −1% • Tear index: −4%	[17]
	BC (static culture)	CHPTAC (DS = 0.004)	BSBP	Retention of fiber increased from 85% to 95%	Dose of 1%. Increments: • TI: 32% • Young modulus: 67% • Burst index: 30%	[78]
Carboxylation	CNC	TEMPO-mediated oxidation	BKSP	No data	Dose of 0.7%. Increments: • TI: 34% • Tear index: 25% • Air permeability: −12%	[79]
Carboxyme-thylation	CNF	Isopropanol + MCA	BKEP	PCC retention increased up to 90% without retention aids and it keeps constant with retention aids	TI decreased from 8%–24% depending the pretreatment intensity and the use of retention aids Air resistance (Gurley porosity) increased 186%	[80]

ACNF: Anionic cellulose nanofibers; BBF: Bleached birch fiber; BSBP: Bleached sugarcane bagasse pulps; β-CEDEA: β-chloroethyldiethylamine; BKSP: Bleached Kraft softwood pulp; CNCC: Cationic hairy nanocellulose; CCNF: Cationic cellulose nanofibers; CHPTAC: (3-Chloro-2-hydroxypropyl)- trimethylammonium chloride; DS: Degree of substitution; DTPA: Diethylenetriamine pentaacetic acid; GCC: Ground calcium carbonate; GT: (2-Hydrazinyl-2-oxoethyl)-trimethylazanium chloride; GTMAC: Glycidyltrimethylammonium chloride; MCA: Monochloroacetic acid; OCC: Old corrugated containerboard; Raisacat-reagent: 73% glycidyl trialkylammonium chloride; poly-DADMAC: Poly-(diallyldimethylammonium chloride); TEA: Tensile energy absorption; TMP: Thermomechanical pulp. * Type of pulp used in the papermaking tests.
